# Use of propensity score methods to address adverse events associated with the storage time of blood in an obstetric population: a comparison of methods

**DOI:** 10.1186/s13104-016-2169-1

**Published:** 2016-07-26

**Authors:** Jillian A. Patterson, Elizabeth A. Stuart, Jane B. Ford

**Affiliations:** 1 Clinical and Population Perinatal Health Research, Kolling Institute, Northern Sydney Local Health District, St Leonards, NSW 2065 Australia; 2 Sydney Medical School Northern, University of Sydney, Sydney, Australia; 3 Department of Mental Health, Department of Biostatistics, Department of Health Policy and Management, Johns Hopkins Bloomberg School of Public Health, Baltimore, MD USA

**Keywords:** Propensity score methods, Blood transfusion, Observational study, Obstetrics

## Abstract

**Background:**

A recent topic of interest in the blood transfusion literature is the existence of adverse effects of transfusing red cells towards the end of their storage life. This interest has been sparked by conflicting results in observational studies, however a number of methodological difficulties with these studies have been noted. One potential strategy to address these difficulties is the use of propensity scores, of which there are a number of possible methods. This study aims to compare the traditional methods for binary exposures with more recently developed generalised propensity score methods.

**Methods:**

Data were obtained from probabilistically linked hospital, births and blood bank databases for all women giving birth from 23 weeks gestation in New South Wales, Australia, between July 2006 and December 2010 with complete information on the birth admission and blood issued. Analysis was restricted to women who received 1–4 units of red cells. Three different propensity score methods (for binary, ordinal and continuous exposures) were compared, using each of four different approaches to estimating the effect (matching, stratifying, weighting and adjusting by the propensity score). Each method was used to determine the effect of blood storage time on rates of severe morbidity and readmission or transfer.

**Results:**

Data were available for 2990 deliveries to women receiving 1–4 units of red cells. The rate of severe maternal morbidity was 3.7 %, and of readmission or transfer was 14.4 %. There was no association between blood storage time and rates of severe morbidity or readmission irrespective of the approach used. There was no single optimal propensity score method; the approaches differed in their ease of implementation and interpretation.

**Conclusions:**

Within an obstetric population, there was no evidence of an increase in adverse events following transfusion of older blood. Propensity score methods provide a useful tool for addressing the question of adverse events with increasing storage time of blood, as these methods avoid many of the pitfalls of previous studies. In particular, generalised propensity scores can be used in situations where the exposure is not binary.

## Background

A common debate in the blood transfusion literature surrounds the risks of adverse outcomes associated with transfusion of older blood [1]. One reason for debate is the large number of conflicting observational studies, of varying quality, which attempt to infer causality without an appropriate experimental design. Evidence from randomized trials is limited, due to the complexity, time and costs involved in performing such experiments. Determining whether there is an independent risk of adverse outcomes following transfusion of older blood can be difficult in an observational setting. The age of the blood that a patient receives varies due to blood bank inventory management processes, blood type, number of transfusions, and time of year [2–4]. These factors may also affect outcomes, and so need to be considered in any analysis. In addition, observational studies of the effect of age of blood on outcomes are prone to a number of confounding factors, including the need to untangle any adverse outcomes due to receiving older blood, from adverse outcomes which result from the condition requiring the transfusion (confounding by indication) [5]. Patients receiving greater numbers of transfusions are also more likely to receive older blood [5]. In addition, there are difficulties in defining the age of blood received, where more than one pack is transfused [6]. Many studies to date have focused on a binary exposure of ‘fresh’ or ‘old’ blood, where there is also the important consideration of what cutpoint to use. Although there are changes that occur in blood as it is stored (termed the ‘storage lesion’), [7] there is no biologically intuitive cutpoint where the build-up of changes would be expected to have an effect, and so cutpoints are somewhat arbitrary, and may not provide sufficient distinction between patients receiving fresh and old blood.

The use of propensity scores has the potential to reduce the effect of confounders on associations between outcomes and age of blood. Propensity score methods have been developed to enable conclusions about causality to be drawn from observational data [8]. Propensity score methods involve the development of a model of the probability of a patient to have received the particular treatment/exposure they received, based on their observed covariates. Under several assumptions [9], patients with similar propensity scores can be considered to have the same likelihood of exposure, and so the average difference in outcome for patients with the same propensity score, but different exposure, can be interpreted as being due to the exposure. The most important assumption for this approach is that treatment/exposure assignment is independent of the outcome given the observed covariates. Propensity scores are most commonly applied to binary exposures, which are less applicable for considering age of blood. While applications of propensity score methodology to ordinal and continuous exposures are less common, methods have been proposed [9, 10], however their use has predominantly been in disciplines other than medicine [11–14]. Applications vary not only in the methods of construction of the propensity score or scores, but also in the approaches to using the score for matching, stratification, weighting or in regression adjustment [8, 15, 16].

Researchers typically perform a single propensity score analysis for a given study, meaning that the performance of the different applications in a real-life situation cannot be compared, although Brookhart et al. [17] perform such a comparison considering only the different approaches to estimating the effect using a binary propensity score. Understanding the differences between the varying propensity score applications in an applied context gives researchers the opportunity to select the application best suited to their research question. Our paper explores the application of three different methods of constructing the propensity score (binary, ordinal and continuous exposures) combined with four approaches to estimating the effect (matching, stratification, weighting and regression adjustment) to the problem of adverse outcomes after transfusion of older blood in a maternity population, focusing on differences in results and implementation.

## Methods

The study population was all women giving birth from 23 weeks gestation in New South Wales, Australia, between July 2006 and December 2010, with complete information on the birth admission and blood issued. Only women receiving from 1 to 4 transfusions were selected to create a group of relatively homogenous risk (by excluding women with massive haemorrhage). The data for this study come from five sources: the New South Wales (NSW) Perinatal Data Collection (‘birth data’); the Admitted Patients Data Collection (‘hospital data’), Clinical Excellence Commission Red Cell Utilisation Database (‘Red Cell data’) and the Australian Red Cross Blood Service (‘Red Cross data’), and the NSW Registry of Births, Deaths and Marriages death registrations (‘deaths data’). These datasets have been described elsewhere [18]. The birth data contains pregnancy, labour and delivery data for women giving birth in NSW, and the hospital data contains data on diagnoses and medical procedures (including transfusion) for all hospital admissions. The Red Cell and Red Cross data together contain information on all blood packs issued from hospital pathology laboratories, including collection date and issue date, from which age of blood at transfusion can be derived. Fact of death was established from the deaths data.

The outcomes considered were readmission to the same or another hospital within 6 weeks of birth, and severe maternal morbidity. Transfers from the delivery hospital were considered a readmission. Severe maternal morbidity included a diagnosis of one or more of sepsis, thromboembolic events, organ dysfunction, shock, cardiac arrest, cerebral oedema, coma, cerebral-vascular accident, assisted ventilation, or dialysis within 6 weeks of delivery, or death (within 12 months). Potential confounders considered were parity, plurality, antepartum or postpartum haemorrhage, gestational diabetes, pregnancy hypertension, maternal age, bleeding or platelet disorders, number of transfusions, month and year of admission, blood type of blood, hospital, hospital level, and leucodepletion.

The age of blood was defined as the age (time between collection and issue of blood pack from the blood bank) of the oldest blood a patient received within the delivery admission. Three methods of constructing the propensity scores were considered: using a binary exposure (splitting age of blood at the median of the maximum age of blood transfused), using quartiles of the maximum age of blood transfused (ordinal exposure), and using the maximum age of blood transfused as a continuous exposure. In each case, the propensity score model was developed using binary logistic, ordinal logistic or linear regression models as appropriate, considering both supply and maternal factors as possible confounders. Models were built using an iterative approach, whereby a model was proposed, balance across covariates assessed, and then the model refined to promote balance. Interactions were included where they improved balance on the propensity score. Balance was assessed by dividing the population into quintiles based on the propensity score, and comparing the proportions of women receiving older vs fresher blood (binary case), or proportions within quintiles of actual age of blood received (ordinal and continuous cases). The application of the four approaches to incorporating the propensity scores differed for each method, and are explained in more detail below. For the purpose of comparison, results are presented as the rate of adverse outcomes with 95 % confidence intervals in each case.

Statistical analyses were performed in SAS (9.3) and R (3.1.0).

## Ethical approvals

This study was approved by the NSW Population and Health Services Research Ethics Committee.

### Method 1: binary propensity score

An arbitrary cutpoint of 22 days (the median age of the oldest blood transfused) was used to divide patients into groups having received any blood >22 days or not. The mean or median are commonly chosen cutpoint in age of blood studies [4, 19, 20], to increase the power of the analysis [5]. Logistic regression was used to construct the propensity score. In order to avoid extrapolating findings beyond the range supported by the data, overlap of cases, the “common support” was assessed by plotting the distribution of propensity scores by older/fresher blood. Cases outside of the common support were excluded to remove patients where the overall treatment effect may be unreliably estimated. This was not needed when matching on propensity scores within a caliper, as the matching process selects only similar cases. A summary of methods for binary propensity scores can be found in Williamson et al. [21] with relevant details outlined below.

#### Matching

Greedy one to one matching without replacement was used to match those receiving older blood to those receiving blood ≤22 days having a similar propensity score. Matches were restricted such that a woman receiving older blood could only be matched to a woman receiving fresher blood whose propensity score was within ±0.05 (the caliper). The rate of adverse outcomes in each group was compared.

#### Stratification

The sample was divided into strata based on quintiles on the basis of the propensity score, and the rate of each adverse outcome calculated within each stratum, and estimates weighted by stratum size summarized over strata.

#### Weighting

Inverse probability of treatment weights were calculated as the inverse of the propensity score for those receiving older blood, and the inverse of 1 minus the propensity score for those receiving fresher blood. These weights were multiplied by the marginal probability of receiving/not receiving older blood for those receiving and not receiving older blood. This stabilization results in the weighted sample size being the same as the original sample size, and reduces the variance of the estimates.

#### Regression adjustment

Logistic regression was used to calculate the odds of adverse outcomes for those receiving blood >22 days, including propensity score in the model. This was used to calculate the predicted adverse outcome rate for those receiving older and fresh blood.

### Method 2: generalized propensity score-ordinal exposure

Women were divided into quartiles (≤15, 16–22, 23–30, 31 days or greater) based on the age of the oldest blood received, with ordinal logistic regression used to model the probability of receiving blood belonging to each age quartile. It has been suggested that where an ordinal logistic regression is appropriate for the data, a single score can be developed for each patient [22, 23]. The linear predictor part of the model is taken as a balancing score, which balances covariates across quartiles [10]. This method results in a single balancing score, and four propensity scores (the probability of belonging to each quartile).

#### Matching

Following the work of Lu et al. [22, 24] and using matching algorithms available in R (nbpMatching) [24] we created matched pairs of subjects where the subjects had similar balancing scores, but different actual age of blood (quartile) received. In matching, preference is given to pairs with the greater difference in quartile (i.e. a patient receiving blood in the first quartile would match to a patient in the third or fourth quartile in preference to one in the second). Within each pair, the patient belonging to the higher quartile was considered to have received ‘older’ blood. The rates of adverse outcome compared for those receiving older vs fresher blood.

#### Stratification

Strata were created by dividing patients into quintiles based on their balancing score. Logistic regression, stratified by balancing score strata, was used to obtain estimated probabilities of adverse outcomes for each quartile and strata. Stratum specific estimates were combined to assess the effect of age of blood quartiles on adverse outcomes.

#### Weighting

Inverse probability of treatment weights were defined as the inverse of the propensity score for the quartile of age of blood received, divided by the marginal probability of that quartile [25]. These weights were applied to estimate the adverse outcome rates.

#### Regression

A logistic regression model including the propensity score and quartile of age of blood, with polynomial terms up to degree 4, was used to examine the relationship between age of blood and adverse outcomes. The model was developed using the actual propensity score and quartile of age of blood received, and then the probability of adverse outcome at each age of blood quartile for each patient calculated using this model and the estimated propensity scores for unobserved quartiles, giving the expected proportion of adverse outcomes for each quartile. Confidence intervals were calculated using 1000 bootstrap samples [9, 12].

### Method 3: generalized propensity score-continuous exposure

A linear regression model was built to predict age of blood received (as a continuous variable), considering supply and maternal factors. The analysis followed the method outlined above for quartiles, using each day of age of blood (days 1–42), instead of quartiles, and using the predicted age of blood as a balancing score. The assumption of constant variance on the multiple linear regression used to construct the propensity score was necessary to create a scalar balancing score and appeared reasonable. The regression model was built considering age of blood as a continuous variable [12]. Rates of adverse events were calculated for each approach, summarized by decile of age of blood.

## Results

Data were available for 2990 deliveries to women receiving 1–4 bags of blood. The median age of the oldest pack of blood transfused to each woman was 22 days. The rate of severe morbidity was 3.7 % (N = 111) and of readmission/transfer was 14.4 % (N = 430).

### Method 1: binary propensity score

Of the 1424 women receiving older blood, 1018 (71 %) were matched to a woman receiving ≤22 day old blood. After matching the rate of severe adverse outcome was 4.2 % (95 % CI 3.1, 5.7) for those receiving fresher blood, and 3.1 % (2.1, 4.3) for those receiving older blood, with an average difference in age of blood of 14.5 days. Removing subjects outside the common support, there were 1412 patients receiving older blood (>22 days) and 1535 receiving fresher blood. The rates of severe morbidity after stratification, weighting and regression adjustment ranged from 3.8 to 3.9 % for fresher blood and 3.0–3.4 % for older blood (Table 1), and for readmission/transfer were from 14.1 to 14.9 % for fresher blood and 13.6–14.7 % for older blood. When considering severe morbidity, each method showed lower rates in the groups receiving older blood, although differences were small and not statistically significant. When considering readmission and transfer this pattern was reflected across matching, stratification and weighting approaches, but not regression adjustment. Regression adjustment was associated with the narrowest confidence intervals, and very similar estimates were obtained for regression and stratification.

**Table 1:** Rate of adverse outcomes following transfusion with fresher or older blood, results from binary propensity score analyses

	Crude rate (95 % CI)	Matched rate (95 % CI)	Stratified rate (95 % CI)	Weighted rate (95 % CI)	Regression rate (95 % CI)
Severe morbidity					
Fresher	4.02 (3.15, 5.12)	4.22 (3.14, 5.65)	3.94 (2.82, 4.84)	3.76 (2.92, 4.82)	3.9 (3.09, 4.72)
Older	3.37 (2.54, 4.45)	3.05 (2.14, 4.30)	3.4 (1.89, 4.92)	2.98 (2.20, 4.02)	3.4 (2.72, 4.08)
Readmission/transfer					
Fresher	14.18 (12.53, 15.99)	14.54 (12.50, 16.84)	14.44 (12.75, 16.14)	14.88 (13.21, 16.72)	14.44 (12.76, 16.13)
Older	14.61 (12.87, 16.54)	14.34 (12.32, 16.63)	14.67 (11.79, 17.56)	13.58 (11.88, 15.48)	14.67 (13.00, 16.33)

### Method 2: generalised propensity score-ordinal exposure

Women were divided into four groups (≤15, 16–22, 23–30, 31 days or greater) based on the age of the oldest blood received. There were 1472 matched pairs created (N = 2944, 98.5 %). The average difference in age of blood received between those receiving older blood and those receiving fresher blood was 12.1 days. After excluding patients outside the common support there were 2860 remaining for analysis. The rates of severe morbidity ranged from 3.5 to 4.9 % for fresher blood, and 3.1–3.8 % for older blood (Table 2), and for readmission/transfer were from 13.4 to 14.9 % for fresher blood, and 13.0–15.2 % for older blood. There were only small differences in outcome rates between quartiles across the different methods, with the middle quartiles tending to have lower morbidity rates.

**Table 2:** Rate of adverse outcomes following transfusion with fresher or older blood, results from ordinal propensity score analyses

	Crude rate (95 % CI)	Matched rate (95 % CI)	Stratified rate (95 % CI)	Weighted rate (95 % CI)	Regression rate (95 % CI)
Severe morbidity					
Freshest	4.23 (2.99, 5.92)	3.53 (2.70, 4.61)	3.74 (1.27, 6.2)	4.92 (3.57, 6.75)	3.6 (2.59, 4.92)
2nd quartile	3.83 (2.69, 5.41)		3.9 (2.44, 5.37)	3.81 (2.64, 5.44)	3.4 (2.6, 4.09)
3rd quartile	2.98 (1.97, 4.46)		3.05 (1.84, 4.25)	2.93 (1.90, 4.45)	3.53 (2.82, 4.46)
Oldest	3.83 (2.58, 5.61)	3.60 (2.76, 4.69)	3.42 (2.01, 4.82)	3.06 (1.95, 4.74)	4.2 (2.76, 6.15)
Readmission/transfer					
Freshest	14.27 (11.95, 16.95)	14.54 (12.83, 16.43)	14.94 (10.01, 19.87)	14.84 (12.44, 17.59)	13.37 (11.35, 15.92)
2nd quartile	14.09 (11.86, 16.67)		14.11 (11.37, 16.84)	13.66 (11.38, 16.31)	13.65 (13.12, 16.05)
3rd quartile	14.14 (11.85, 16.78)		14.17 (11.75, 16.6)	13.36 (11.07, 16.04)	13.3 (12.68, 15.74)
Oldest	15.16 (12.61, 18.12)	14.47 (12.76, 16.36)	14.96 (12.18, 17.75)	14.51 (11.97, 17.47)	12.97 (10.92, 15.75)

### Method 3: generalised propensity score-continuous exposure

There were 1490 matched pairs created (N = 2980, 99.7 %). The average difference in age of blood received between those receiving older blood and those receiving fresher blood was 12.3 days. After excluding patients outside the common support, there were N = 2756 available for the remaining analyses. There was no difference in rates of severe morbidity or readmission/transfer across deciles of age of blood (Table 3). With some exceptions, rates across deciles tended to be similar for the stratification and weighting approaches, where they differed, the weighting values tended to be more extreme. Both sets of rates tended to ‘jump around’, with little trend evident between deciles. The regression rates however were smoother between deciles, and less extreme than either weighting or stratification, except for the highest and lowest deciles.

**Table 3:** Rate of adverse outcomes following transfusion with fresher or older blood, results from generalised propensity score analyses

	Crude rate (95 % CI)	Matched rate (95 % CI)	Stratified rate (95 % CI)	Weighted rate (95 % CI)	Regression rate (95 % CI)
Severe morbidity					
Freshest	5.50 (3.41, 8.69)	4.03 (3.13, 5.16)	5.80 (3.12, 8.48)	5.11 (3.00, 8.45)	5.97 (2.02, 18.87)
2nd decile	3.14 (1.70, 5.61)		2.74 (1.22, 4.26)	4.45 (2.64,7.32)	4.22 (2.25, 7.94)
3rd decile	2.82 (1.41,5.35)		2.31 (0.63, 3.99)	2.11 (0.89, 4.52)	3.64 (2.17, 6.14)
4th decile	4.87 (2.64,8.60)		5.09 (2.36, 7.82)	5.64 (3.18, 9.66)	3.54 (2.29, 5.46)
5th decile	4.14 (2.47,6.78)		4.27 (2.2, 6.35)	4.16 (2.46, 6.85)	3.59 (2.41, 5.32)
6th decile	4.21 (2.41, 7.13)		4.11 (1.74, 6.48)	4.11 (2.29, 7.12)	3.65 (2.44, 5.44)
7th decile	2.76 (1.31, 5.44)		2.96 (0.66, 5.27)	2.71 (1.24, 5.52)	3.6 (2.21, 5.87)
8th decile	2.83 (1.26, 5.85)		2.63 (0.17, 5.09)	3.83 (1.90, 7.28)	3.38 (1.79, 6.54)
9th decile	3.72 (2.07, 6.45)		3.1 (−2.40, 8.6)	2.10 (0.87, 4.59)	2.86 (1.23, 7.16)
Oldest	3.14 (1.49, 6.17)	3.42 (2.61, 4.48)	1.98 (−2.17, 6.13)	1.36 (0.23, 4.30)	1.83 (0.47, 8.54)
Readmission/transfer					
Freshest	14.56 (11.04, 18.95)	14.16 (12.48, 16.03)	14.86 (10.72, 18.99)	19.33 (15.08, 24.41)	12 (6.40, 23.47)
2nd decile	15.14 (11.75, 19.29)		14.94 (11.20, 18.68)	14.68 (11.24, 18.94)	13.96 (10.23, 18.99)
3rd decile	12.23 (9.05, 16.30)		12.54 (9.03, 16.05)	13.66 (10.22, 18.01)	14.18 (11.03, 18.12)
4th decile	13.27 (9.42, 18.36)		13.42 (9.01, 17.84)	13.75 (9.74, 19.02)	14.29 (11.6, 17.49)
5th decile	15.19 (11.84, 19.27)		15.39 (11.71, 19.06)	15.31 (11.90, 19.47)	14.48 (11.99, 17.39)
6th decile	15.21 (11.61, 19.66)		14.73 (10.50, 18.95)	14.52 (10.91, 19.05)	14.83 (12.26, 17.84)
7th decile	13.45 (9.97, 17.88)		14.07 (9.51, 18.64)	13.72 (10.10, 18.37)	15.19 (12.12, 18.89)
8th decile	15.79 (11.74, 20.89)		16.23 (9.92, 22.54)	18.19 (13.70, 23.74)	15.35 (11.55, 20.19)
9th decile	14.24 (10.83, 18.50)		14.48 (5.33, 23.63)	14.63 (11.00, 19.18)	15.08 (10.54, 21.4)
Oldest	14.51 (10.68, 19.39)	14.63 (12.93, 16.52)	13.86 (1.81, 25.91)	10.74 (7.12, 15.82)	13.29 (7.70, 22.62)

## Discussion

This study found no adverse effect of transfusion of older blood on maternal outcomes. Twelve different analyses using three methods of constructing the propensity score and four approaches to applying it were performed for each adverse outcome, with a high degree of consistency across methods. None of the methods considered showed a beneficial or detrimental effect of older blood on patient outcomes. The obstetric population provides an ideal population in which to study the effect of age of transfused blood on patient outcomes, as in this population patients are generally young and otherwise healthy [26]. A more complete discussion of the effect of age of blood in an obstetric population can be found in Patterson et al. [26]. This finding of no effect of age of blood is consistent with several studies amongst lower risk patients [27, 28], however in some specific populations age of blood has been shown to affect outcomes [1, 29–32]. The adequacy of methods used in these studies to address confounding has been questioned [5]. Use of propensity score methods enabled us to separate the effect of older blood from confounders, particularly the number of units transfused. Consideration of propensity scores for ordinal and continuous exposures allowed us to move away from the need to dichotomise age of blood, which although commonly used, has little physiological justification [5]. Different propensity score methods were used, resulting in different estimates of effect, with each method and approach having different benefits and drawbacks.

Propensity scores are becoming more widely used, and have a number of advantages over other methods. In particular, matching on a propensity score for a binary exposure creates a situation similar to the baseline balance achieved in a randomized trial (on measured confounders), and so is accessible for clinicians [8]. It is also possible to assess the balance created by the propensity score method [14, 33], and to exclude subjects where the results are unlikely to apply [33]. Another key benefit lies in the two step process of analysis, where the modeling process (constructing the propensity score) is conducted separately to the analysis of results, maintaining a level of objectivity [14, 33]. As noted by Zanutto et al. an added benefit of this approach, used in this study, is that the same propensity score can be used across multiple outcomes [14]. In cases where the outcome is rare, but the exposure is common, traditional regression models are not able to fully model confounding, however propensity scores are able to adjust for more confounders [8, 23].

Across the three methods of constructing the propensity score (using binary, ordinal or continuous exposures) used in our study, there were differences in the performance of the different approaches. The difference in age of blood received between pairs decreased when using the generalized propensity score compared with the binary and ordinal score approaches, however matching was more successful (greater proportion of patients matched) when using the generalized propensity score than with the ordinal and binary methods. This is due to the smaller number of potential matches excluded due to having the same value of the exposure. The impact of this can be seen in narrower confidence intervals compared with other methods when using ordinal or generalized propensity score methods. In contrast, more patients were excluded when using the generalized propensity score and ordinal propensity score methods for being outside of the ‘common support’, where a patient is deemed to have received an unusual treatment given their covariate pattern.

Within the binary propensity score method, stratification, weighting and regression produce similar estimates of effect, with the narrowest confidence intervals associated with the regression estimates. The confidence intervals associated with matching were wider, reflecting the smaller sample size used in this approach. With the ordinal model, stratification tended to give the widest confidence intervals, with matching and regression producing narrower intervals. However, using the generalized propensity score, the greatest uncertainty was associated with the regression model, reflecting the variability both in the propensity score and the modeling process. Stratification typically performed better in terms of reduced variability. Patterns of estimates obtained via stratification and weighting were similar when using the ordinal and generalized propensity scores, but differed from the results obtained from the regression based approached. The regression approaches impose a degree of smoothness between estimates of adjoining categories which the other estimates are unable to account for. This additional smoothness was more noticeable with the generalized propensity score than with the quartiles. Given the known ordering of quartiles and deciles, it seems beneficial to incorporate this knowledge in the effect estimates.

The development and assessment of the propensity score models was most straightforward for the binary propensity score, as this resulted in two groups that could easily be compared to assess balance. In the more complex methods, both the exposure and propensity score need to be stratified, and patients within stratum compared in order to determine if balance has been obtained. Here we used quintiles of propensity score and observed age of blood, resulting in 25 strata. This difficulty carries over to the interpretation of results. It is possible to obtain effect estimates, odds ratios and other measures of effect for the binary and ordinal propensity score models, but for the generalized model, with the exception of matching, the relationship between outcome and exposure is difficult to summarise, and may be better captured graphically [12, 34].

The relative merits of the different methods for a binary exposure have been discussed elsewhere [17] and carry across to the more complex designs with several exceptions. Weighting and stratification in the case of non-binary exposures represent only a small increase in complexity compared with the binary case. Weighting methods, while easily applied in the binary propensity score case, do not exploit the extra information available in the case of ordinal or continuous data, [34] and are more difficult in cases with a truly continuous outcome [35]. Weighting is also difficult when more than one propensity score has been used for each subject [22, 34, 36]. Stratification however is easy to apply in cases of one or more propensity score, [14] and retains the ease of interpretation that is present with binary scores [37].

Applications of matching and regression adjustment methods are somewhat more complicated in the case of non-binary exposures. While matching is intuitive in the case of binary exposures, in some sense replicating the setup of a controlled trial (although only ensuring groups are the same based on observed covariates), with more than 2 exposures matching is less intuitive. In the continuous case, the comparison of ‘older vs fresher’ blood is conducted without defining ‘older’ or ‘fresher’ across the sample. It is possible that there could be a matched pair with ages of blood 7 and 10 days, and another pair with ages 32 and 36 days. In this case, the patients receiving blood of 7 and 32 days would be included as receiving ‘fresher’ blood, even though the difference in age of blood received is considerable. It has been argued that matching can be helpful, even when a null result is returned, in that it can be interpreted as follows: ‘considering the greatest possible differences between age of blood received, there was no effect of age of blood on outcomes, hence no effect would be expected at smaller differences’ [22]. Newer techniques allow for matching in categorical outcomes, where one subject from the group with the smallest number of subjects is matched to one or more subjects in the remaining groups, and the matched sample used in regression analysis or similar [36, 38]. The matching algorithm needed for matching with more than two exposures, although available, makes this method less accessible than stratification, weighting and regression which can be performed using traditional software. For this reason, it was considered beyond the scope of this paper [14]. Regression adjustment in the non-binary case can be performed in normal statistical software, but requires several additional steps. In particular, parameters obtained from the regression equation are not able to be interpreted directly, but need to be averaged over the distribution propensity scores evaluated at that dose [34].

It is important to note that when using matching techniques, the whole population is not included in the analysis, and so reported rates reflect the incidence of outcomes only in those women who were able to be matched to a woman in the other arm. With other methods, where ‘common-support’ criteria are imposed, the study populations are likewise limited to those women who similar in terms of likelihood of exposure at varying treatment levels. In some cases, when a large number of women have been excluded, this may be quite different from the population rate [33]. A comparison of included and excluded cases would be important in practice to aid in the interpretation of results and generalisation to the wider population.

This study explored the application of different propensity score methods to the effect of age of blood on adverse outcomes. We used a large sample, with information on many potential confounders, and considered two outcomes: one with low incidence and one more common. A possible limitation of this study is that any unmeasured confounders such as hospital policies or clinician practice of preferentially transfusing fresher blood to sicker patients would not be adjusted for by the propensity score (regardless of method used), and may introduce bias into the result. Adjustment by hospital may somewhat offset such bias, but other unknown confounders may also affect results. In addition, for simplicity we only used one method of checking for balance, although several methods have been proposed and are used in practice, [9, 10, 22, 39] and these methods may be more appropriate for particular approaches, such as the use of standardized mean differences [39] for binary matching. In practice, the balance method chosen should relate to the methods used in the analysis [33].

## Conclusions

Propensity score methods are useful for the analysis of observational data around the age of blood transfused, and allow causal inference from such data. These methods are able to account for differences in number of blood packs transfused and other confounders that influence both the age of blood transfused and potential adverse outcomes. Although less intuitive than their binary exposure counterparts, propensity score methods for ordinal and continuous exposures are feasible, able to be implemented with standard software (using packages available online), and better reflect the underlying mechanism of age of blood. These methods should be considered in similar studies where it is not appropriate to dichotomise an exposure, and where the outcome is sufficiently rare to limit the utility of regression modeling. Each of the three methods (binary, ordinal and continuous exposure) produced slightly different estimates of effect, but found no significant relationship between age of blood transfused and adverse outcomes.

## Abbreviation

NSW: New South Wales
